# Machine Learning for Cancer Immunotherapies Based on Epitope Recognition by T Cell Receptors

**DOI:** 10.3389/fgene.2019.01141

**Published:** 2019-11-19

**Authors:** Anja Mösch, Silke Raffegerst, Manon Weis, Dolores J. Schendel, Dmitrij Frishman

**Affiliations:** ^1^Department of Bioinformatics, Wissenschaftszentrum Weihenstephan, Technische Universität München, Freising, Germany; ^2^Medigene Immunotherapies GmbH, a subsidiary of Medigene AG, Planegg, Germany

**Keywords:** cancer immunotherapy, T cell receptor, neoepitope, neoantigen, cross-reactivity, MHC binding affinity prediction

## Abstract

In the last years, immunotherapies have shown tremendous success as treatments for multiple types of cancer. However, there are still many obstacles to overcome in order to increase response rates and identify effective therapies for every individual patient. Since there are many possibilities to boost a patient’s immune response against a tumor and not all can be covered, this review is focused on T cell receptor-mediated therapies. CD8^+^ T cells can detect and destroy malignant cells by binding to peptides presented on cell surfaces by MHC (major histocompatibility complex) class I molecules. CD4^+^ T cells can also mediate powerful immune responses but their peptide recognition by MHC class II molecules is more complex, which is why the attention has been focused on CD8^+^ T cells. Therapies based on the power of T cells can, on the one hand, enhance T cell recognition by introducing TCRs that preferentially direct T cells to tumor sites (so called TCR-T therapy) or through vaccination to induce T cells *in vivo*. On the other hand, T cell activity can be improved by immune checkpoint inhibition or other means that help create a microenvironment favorable for cytotoxic T cell activity. The manifold ways in which the immune system and cancer interact with each other require not only the use of large omics datasets from gene, to transcript, to protein, and to peptide but also make the application of machine learning methods inevitable. Currently, discovering and selecting suitable TCRs is a very costly and work intensive *in vitro* process. To facilitate this process and to additionally allow for highly personalized therapies that can simultaneously target multiple patient-specific antigens, especially neoepitopes, breakthrough computational methods for predicting antigen presentation and TCR binding are urgently required. Particularly, potential cross-reactivity is a major consideration since off-target toxicity can pose a major threat to patient safety. The current speed at which not only datasets grow and are made available to the public, but also at which new machine learning methods evolve, is assuring that computational approaches will be able to help to solve problems that immunotherapies are still facing.

## Introduction

**Figure 1:**
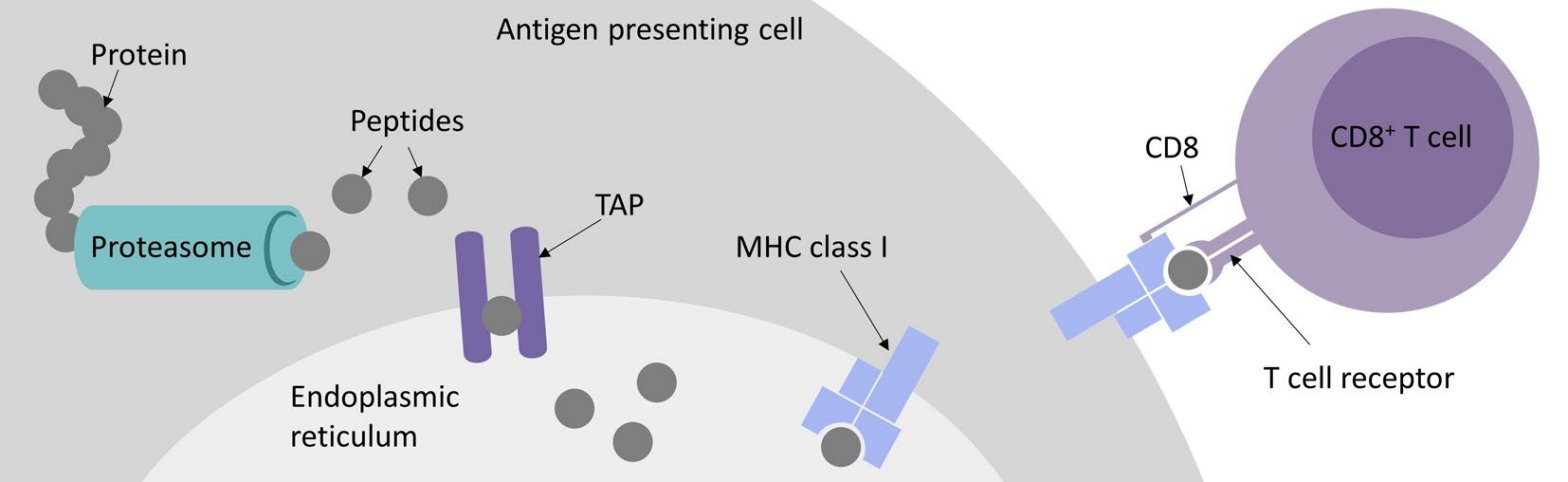
Major histocompatibility complex (MHC) class I antigen presentation pathway for peptides recognized by CD8^+^ cytotoxic T cells.

Immunotherapies have gained more and more importance over the last decades. Checkpoint inhibitors mainly targeting PD1/PDL1 and CTLA4 and personalized cancer vaccines (Gubin et al., 2014; Ott et al., 2017; Sahin et al., 2017) have been and still are heavily investigated in clinical trials. Both depend on patient individual tumor-specific mutations enabling the boost of a cancer-specific T cell-mediated immune response (Snyder et al., 2014; Rizvi et al., 2015; Łuksza et al., 2017). A more direct approach utilizes the adoptive transfer of a patient’s autologous T cells, either genetically modified with a transgenic chimeric antigen receptor (CAR) or T cell receptor (TCR). For CAR-T cell as well as TCR-T cell therapy a defined target, the epitope, needs to be identified. CARs, carrying the functional antigen-binding domain of an antibody, recognize three-dimensional peptide structures on the surface of a cell (Sadelain et al., 2013). By contrast, TCRs recognize predominantly linear peptides presented by the major histocompatibility complex (MHC) called human leucocyte antigen (HLA) in humans. For MHC class I presentation and thus CD8^+^ T cell detection, these peptides come from proteins that are intracellularly processed by either the constitutive proteasome or the IFNγ induced immunoproteasome (Griffin et al., 1998; Neefjes et al., 2011). After cleavage, the peptides are transported to the endoplasmic reticulum (ER) by the transporter associated with antigen processing (TAP) complex, where they are loaded onto MHC class I molecules. The peptide-MHCs (pMHCs) are shuttled to the cell surface where they can potentially be recognized by CD8^+^ cytotoxic T cells, either naturally carrying or engineered to bear a pMHC-specific TCR (see **Figure 1**). However, there are more than 16,000 different alleles for *HLA-A*, -*B*, and -*C* genes, which bind and present different epitopes (Robinson et al., 2015). Besides MHC class I mediated CD8^+^ cytotoxic T cell responses, MHC class II bound peptides can induce CD4^+^ T cell responses that are also reported to play an important role in tumor detection and elimination (Nielsen et al., 2010; Linnemann et al., 2014; Kreiter et al., 2015; Andreatta et al., 2017; Veatch et al., 2018).

A wide spectrum of bioinformatics tools exists for modeling all steps of the MHC class I antigen presentation pathway, including proteasomal cleavage, translocation of the peptides to the ER by TAP, peptide binding to the MHC molecules, and TCR recognition. The overarching goal of these efforts is to enhance our understanding of how T cell epitopes are selected from a virtually unlimited number of short peptides that can be proteolytically generated from the human proteome. The origin of these T cell epitopes can be naturally occurring proteins or peptides derived from somatic mutations. For personalized cancer immunotherapy, these patient- and tumor-specific mutations are usually separately assessed for each patient by exome sequencing, mutation detection and peptide binding prediction (Robbins et al., 2013; Blankenstein et al., 2015; Schumacher and Schreiber, 2015). Predicting these so called neoepitopes or neoantigens is a prevailing challenge for computational methods for immunotherapy and essential for a high-throughput approach to narrow down mutations to be included in vaccines or to be evaluated *in vitro* for T cell recognition, since only very few mutations are truly immunogenic (Yadav et al., 2014; Strønen et al., 2016; Bjerregaard et al., 2017a).

It is also of utmost importance to evaluate potential cross-reactivity of target-candidate epitopes based on various omics data such as proteomics and peptidomics (Haase et al., 2015; Jaravine et al., 2017a; 2017b). However, all existing approaches based on epitope presentation are only a surrogate for T cell recognition, for which no universal and computationally viable approach exists so far, although the first promising results have been published (Jurtz et al., 2018; Ogishi and Yotsuyanagi, 2019). By now, datasets have been generated that allow sequence-based prediction approaches using deep learning (Shugay et al., 2018; Vita et al., 2018).

In this review, we summarize the current state at the development of prediction algorithms and methods for all steps of antigen presentation, evaluate neoepitope prediction approaches, and discuss progress toward sequence-based TCR binding prediction.

## Prediction of T Cell Epitopes

### Proteasomal Cleavage Prediction

In order to develop an accurate prediction algorithm for proteosomal cleavages, a thorough mechanistic understanding of the cutting process is required. The PAProC algorithm by Kuttler et al. (Kuttler et al., 2000) relies on a biologically motivated model, which postulates that proteolytic sites are mostly determined by the local sequence context, generally not further away in the sequence than six amino acid residues. The two residues immediately adjacent to the cut make the greatest contribution to the affinity to the active subunits of the proteasome, while the influence of the other surrounding residues is lower. The recognition model is additive in that the total affinity, which ultimately determines the probability of the cut, is considered to be the sum of all individual contributions. Bioinformatics analyses revealed that the amino acids in the six positions preceding the cut and four positions downstream contain sufficient information to reproduce a training dataset of experimentally determined cleavage motifs of 20S proteasomes by a network-based technique. Keşmir et al. (Keşmir et al., 2002) demonstrated that good results in detecting proteasomal cleavage motifs can be achieved by combining experimental data on degradation by the constitutive proteasome with the sequences of peptides bound by the MHC class I molecules, which may be generated either by the constitutive or by the immunoproteasomes. A neural network trained on such a composite dataset, called NetChop, and an updated version NetChop 3.0 (Nielsen et al., 2005), achieved a reasonable accuracy and also yielded useful insights into cleavage-promoting and inhibiting residues as well as into N-terminal extension of peptides after proteasomal cleavage. A recurrent difficulty in predicting proteasomal cleavage is the lack of experimentally verified noncleavage sites. However, such negative data can be artificially generated by considering internal positions of confirmed MHC ligands or randomly generated sites.

### TAP Binding Prediction

An early study of Daniel et al. (1998), in which the TAP binding affinity for a large number of peptides of length nine was measured by a peptide binding assay, revealed that positions one to three and nine of the 9-mers make the largest contribution to the selectivity of TAP to peptides. An artificial neural network trained on these data was able to predict the IC_50_ values with high accuracy. The study also found that HLA class I molecules differed significantly with respect to TAP affinities of their ligands. The predictive scope was later extended to peptides of arbitrary length using a stabilized matrix approach and a scoring scheme that only considers the first three N-terminal residues and the last C-terminal residue (Peters et al., 2003). Since it has been established that the selectivity of peptide transport by TAP is entirely determined by the peptide-binding step (Gubler et al., 1998), affinity predictions can be equated with translocation likelihood predictions. A number of further machine learning methods for predicting peptide binding to TAP were trained on 9-mer data, which is the typical length of the peptides that will subsequently bind to the MHC complex (Bhasin, 2004; Zhang et al., 2006; Diez-Rivero et al., 2010; Lam et al., 2010).

### Peptide-MHC Binding Prediction

Sequencing of peptides eluted from MHC class I molecules (Falk et al., 1991) as well as mass-spectrometric (MS) (Hunt et al., 1992) and crystallographic (Madden, 1995) evidence revealed common properties of the epitopes, in particular the typical length range of 8–12 residues. Additionally, it showed the existence of MHC allele-specific anchor residues, usually in positions two and nine of the core nonameric segments, as well as auxiliary anchors, where amino acid preferences are less strict (Rammensee et al., 1993).

Starting from the early nineties, efforts were made to collect available information on MHC class I ligands (Brusic et al., 1994; Rammensee et al., 1995; Rammensee et al.,1999) and to predict them using simple motif- and profile-based techniques (Rothbard and Taylor, 1988; Parker et al., 1994; Reche et al., 2002), based on the notion that peptides highly similar in sequence to experimentally characterized ligands will have a higher binding potential than more distantly related peptides and that individual amino acid side chains make independent contributions to the overall binding energy. Machine learning techniques, such as neural networks and hidden Markov models (Bisset and Fierz, 1993; Mamitsuka, 1998; Nielsen et al., 2003) outperform matrix-based methods in predicting peptide binding affinity (Peters et al., 2006; Lin et al., 2008). They are able to deal with peptides of variable length (Lundegaard et al., 2008) and to take into account nonadditive effects, which may arise, e.g., when two amino acids compete for the same site in the peptide-binding groove of the MHC heterodimer. The latest version of the widely used NetMHC algorithm 4.0 (Andreatta and Nielsen, 2016) was trained on many thousands of quantitative affinity measurements for peptides of length 8–11 and the total of 118 MHC class I alleles from human, other primates, and mouse. Neural networks trained on all peptides (allmer networks) significantly outperformed the networks trained on peptides of each individual length separately. The study also suggested specific binding modes for 10- and 11-mers, which are predicted to bulge out of the MHC grove in contrast to 8- and 9-mers, which are strictly linear epitopes. MHCflurry, which relies on affinity measurement and peptide elution MS data, also uses neural networks trained individually for each HLA allele (O’Donnell et al., 2018b). Additionally, it allows users to train networks locally on data of their choice. This can be important especially for cancer immunotherapy applications, since peptide-binding affinity predictions are traditionally focused on viral epitopes.

There is also a growing group of pan-specific methods, including PickPocket (Zhang et al., 2009), NetMHCpan 4.0 (Jurtz et al., 2017), PSSMHCpan (Liu et al., 2017), and ACME (Hu et al., 2019), which take as input both the peptide and the HLA sequence and are able to predict the binding of any peptide to any allele. Most predictions are focused on MHC class I, but there are also methods available for MHC class II, such as NetMHCII 2.3 and NetMHCIIpan 3.2 (Jensen et al., 2018), ProPred (Singh and Raghava, 2001), SMM-align (Nielsen et al., 2007), and NNAlign (Nielsen and Andreatta, 2017), of which the latter also allows to train and use own models, as Garde et al. did for MHC class II prediction using both affinity measurement and MS data (Garde et al., 2019). Many of the aforementioned prediction methods for both MHC class I and II and consensus methods, such as NetMHCcons (Karosiene et al., 2012) and the consensus method by Moutaftsi et al. (Moutaftsi et al., 2006), are integrated into the IEDB epitope analysis resource and can be accessed online (Wang et al., 2010; Fleri et al., 2017; Vita et al., 2018; Dhanda et al., 2019). In addition, combinatory pipelines and frameworks have been published, namely, EpiJen (Doytchinova et al., 2006), NetCTL (Larsen et al., 2007), NetCTLpan (Stranzl et al., 2010), and FRED2 (Schubert et al., 2016), modeling the complete antigen presentation pathway by including proteasomal cleavage and TAP transport predictions.

Epitope presentation, however, is only one step toward T cell recognition. NetMHCstab (Jørgensen et al., 2014) and NetMHCstabpan (Rasmussen et al., 2016) are methods to predict the stability of pMHC complexes, presuming that epitope presentation lasting longer increases the likelihood of T cell recognition and thus immunogenicity. Calis et al. proposed a scoring model to predict true immunogenicity of T cell epitopes (Calis et al., 2013). Despite these efforts, however, true immunogenicity remains far more difficult to predict than mere MHC-binding affinity.

Beyond sequence-based approaches, significant methodological progress has been made in modeling peptide binding to MHC class I molecules on structure level. The diversity of the cognate peptide repertoire and the experimental binding profiles for a particular MHC protein can be accurately captured using both general purpose modeling packages, such as Rosetta (Yanover and Bradley, 2011), and faster specialized methods, such as GradDock (Kyeong et al., 2018), DockTope (Menegatti Rigo et al., 2015), and LYRA (Klausen et al., 2015), of which the latter two are also integrated in the IEDB. Docking experiments are becoming increasingly successful in reproducing crystallographically known peptide-MHC binding geometry (Bordner and Abagyan, 2006; Antunes et al., 2018).

### Immunopeptidomics Data

The recent availability of large-scale immunopeptidomics data allowed to explicitly model peptide length distributions and the interdependence between individual sequence positions, leading to more accurate predictions of naturally presented MHC class I ligands (Gfeller et al., 2018). MS profiling provides novel insights into the antigen processing rules, including the discovery of binding motifs, improved description of proteasomal cleavage signatures, cellular localization and sequence features of peptide source proteins, and better understanding of the role of gene expression, protein abundance and degradation (Bassani-Sternberg et al., 2015; Bassani-Sternberg et al., 2017; Abelin et al., 2017). In particular, Abelin et al. (2017) reported that neural networks trained on MS-derived peptides bound to 16 different HLA alleles outperformed affinity-trained predictors.

For immunogenicity, T cell epitope verification by TCRs or TCR-like antibodies would constitute an ideal dataset to train prediction algorithms (Dolan, 2019), but both approaches are highly dependent on specificity and affinity of TCRs and antibodies used and do not reach the high-throughput efficiency of immunopeptidomics. HLA-peptidomics, which is the MS analysis of MHC-eluted peptides, is the most sophisticated method for high-throughput qualitative and quantitative detection of MHC ligands and thereby of potential T cell epitopes (Hunt et al., 1992; Caron et al., 2011; Hassan et al., 2014; Álvaro-Benito et al.,2018; Freudenmann et al., 2018).

**Figure 2:**
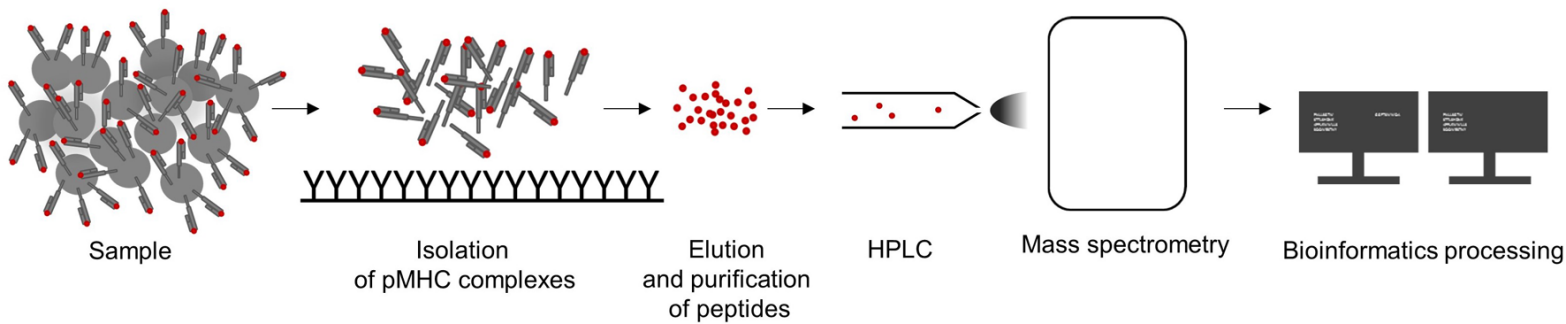
Workflow to analyze of major histocompatibility complex (MHC)-eluted peptides by mass-spectrometric (MS). A sample is lysed, pMHC complexes are captured and peptides are purified by immunoaffinity purification using MHC-specific immobilized antibodies. Eluted peptides are separated by high pressure liquid chromatography (HPLC), analyzed by MS, and the resulting data are computationally processed.

The isolation of pMHC complexes from cell surfaces (Sugawara et al., 1987; Storkus et al., 1993; Bassani-Sternberg et al., 2015; Marino et al., 2019) or out of serum (Ritz et al., 2016; 2017) is the first critical step for a high-quality MS HLA-peptidome analysis. After elution from pMHC complexes, peptides are purified, separated by high pressure liquid chromatography (HPLC), and directly injected and analyzed in a mass spectrometer followed by computational processing of MS spectra data (see **Figure 2**). Successful peptide detection is determined by various factors, such as HLA enrichment, which is dependent on HLA-antibody quality, efficient elution, and physicochemical characteristics of a peptide defined by its amino acid composition. Relevant peptide properties can be mass, hydrophilicity, and hydrophobicity, its ability to be ionized, as well as cysteine content (Gfeller and Bassani-Sternberg, 2018). Therefore, not all peptides are equally likely to be detected by MS but it is difficult to assess how many peptides are missed. Peptide sequences are often determined by tandem MS: a precursor mass spectrum called MS1 spectrum of the eluted peptides is generated and only peptides with high intensities are isolated for fragmentation and analyzed, resulting in a MS2 or MS/MS spectrum. Observed mass spectra are then compared with theoretical mass spectra in general reference databases. Proteogenomic computational pipelines using customized reference datasets also allow the identification of peptides originating from noncanonical and allegedly noncoding reading frames (Laumont and Perreault, 2017; Laumont et al., 2018), unconventional, genomic coding-sequences (Erhard et al., 2018) as well as neoepitopes from somatic alterations (Yadav et al., 2014; Carreno et al., 2015) or intron retentions (Smart et al., 2018). In addition, the generation of customized spectral library databases of high confidence peptides can be used for data-independent acquisition approaches (Ritz et al., 2017), resulting in increased reproducibility and sensitivity.

Peptides are often assigned to the HLA molecule from which they were originally eluted by predicting the binding affinity (Freudenmann et al., 2018; Bilich et al., 2019). For common HLA alleles, usually a sufficient number of peptides are identified as binders, resulting in datasets large enough to train prediction algorithms. However, for less frequent HLA alleles, the pool of identified and correctly assigned peptides is more limited, which leads to variability in performance of prediction techniques depending on the rarity of each HLA allele (O’Donnell et al., 2018b). If MS datasets annotated by binding affinity predictions are used to train machine learning algorithms, a self-amplifying bias is introduced. MS profiling of mono-allelic cells (Giam et al., 2015; Abelin et al., 2017) as well as deconvolution approaches (Bassani-Sternberg and Gfeller, 2016) can circumvent this problem and improve the quality of available training data and prediction performance.

## Immunotherapy-Specific Applications of Epitope Prediction

### Neoepitope Identification

Cancer-specific mutations have been demonstrated to be viable targets for tumor-infiltrating lymphocytes (TILs) enabled by checkpoint inhibitors that block CTLA4 or PD1/PDL1 or by vaccine-induced immune responses (van Rooij et al., 2013; Carreno et al., 2015; Cohen et al., 2015; Gros et al., 2016; McGranahan et al., 2016; Ott et al., 2017; Zacharakis et al., 2018; Hilf et al., 2019). These mutations alter amino acid sequences of proteins and are recognized as so called neoepitopes or neoantigens, with both terms used ambiguously and oftentimes synonymously in the literature. Here, we use the term neoepitopes for epitopes predicted to be presented by a certain MHC and the term neoantigens for confirmed immunogenic mutations. By definition, neoantigens are tumor-specific, which makes them ideal immunotherapy targets, but they are also to a large degree patient-specific. Despite many efforts, only very few shared neoantigens such as KRAS^G12D/V^ or BRAF^V600E^, could be identified, making an off-the-shelf therapy approach hardly feasible (Warren and Holt, 2010; Angelova et al., 2015; Tran et al., 2015; Thorsson et al., 2018). Furthermore, a high individual tumor mutation burden and the ambition to provide personalized medicine for more patients do not allow for testing the immunogenicity of every mutation *in vitro*. Therefore, the current standard procedure for individual patients relies on exome sequencing followed by mutation calling and machine learning-based neoepitope prediction, which represents the main application of pMHC-binding prediction algorithms in the field of cancer immunotherapy. Here, we reviewed more than 70 publications using binding prediction algorithms to identify neoepitopes of which 49, that provided quantifiable data, are shown in **Table 1**. Not all studies stated all steps of their neoepitope selection process, including which algorithm parameters were used, how many neoepitopes were found when applying a threshold or how many and what types of mutation were used for predicting neoepitopes, which makes quantitative evaluation and reproducibility difficult. This is aggravated by the large variance in ratio of predicted neoepitopes per mutation, which is caused by thresholds of varying strictness, the number of features used for filtering, and the approach to counting neoepitopes or neoantigens, i.e., if a mutation was counted only once even if presented by more than one HLA allele or contained in multiple epitopes predicted to be immunogenic. Furthermore, some studies could only experimentally validate a subset of predicted neoepitopes and experimental validation was determined by biological assays of varying sensitivity from MHC-ligand confirmation to ELISPOT assays using patient-specific TILs.

**Table 1:** Publications describing the application of machine learning approaches to neoepitope prediction.

Publication	Indication	Sample type and number	number of HLAs used	Estimated ratio of predicted neoepitopes from mutations	Estimated ratio of experimentally confirmed neoantigens	Number of features	Algorithms
(Segal et al., 2008)	BRCA/CRC	11 patients	1	0.17	N/A	1	NetMHC, SYFPEITHI, BIMAS, RANKPEP
(Castle et al., 2012)	MEL	1 murine cell line	N/S	0.05	0.32^T^	2	NetMHC
(Khalili et al., 2012)	various	312 genes (COSMIC)	57	1.40	N/A	2	NetMHC 3.2
(Robbins et al., 2013)	MEL	3 patients	2	0.18	0.03 ^T^	3	NetMHCpan 2.4
(van Rooij et al., 2013)	MEL	1 patient	4	0.42	<0.01 ^T^	3	NetChop, NetMHC 3.2
(Boegel et al., 2014)	various	167 cancer cell lines	6	0.44	N/A	1	IEDB 2.9
(Duan et al., 2014)	SARC	2 murine tumors	3	0.75	0.56 ^T^	2	NetMHC 3.0
(Snyder et al., 2014)	MEL	64 patients	6	0.42	<0.01 ^T^	3	NetMHC 3.4, RANKPEP, IEDB immunogenicity, CTLPred
(Yadav et al., 2014)	CRC/PRAD	2 murine cell lines	2	0.03	0.02 ^T^	3	NetMHC 3.4
(Angelova et al., 2015)	CRC	552 TCGA patients	6	0.41	N/A	2	NetMHCpan
(Carreno et al., 2015)	MEL	7 samples/3 patients	1	0.04	0.43 ^B^	3	NetMHC 3.4
(Cohen et al., 2015)	MEL	8 patients	2	0.02	0.02 ^T^	2	IEDB
(Rizvi et al., 2015)	NSCLC	34 patients	6	0.62	<0.01 ^T^	2	NetMHC 3.4
(Rooney et al., 2015)	various	4250 TCGA patients	6	0.14	N/A	2	NetMHCpan 2.4
(Tran et al., 2015)	GIC	10 patients	12	0.03	0.21 ^T^	2	NetMHCpan 2.8, NetMHCIIpan 3.0
(Van Allen et al., 2015)	MEL	110 patients	6	1.56	N/A	2	NetMHCpan 2.4
(van Gool et al., 2015)	UCEC	245 TCGA patients	1	0.06	N/A	3	NetMHCpan 2.8
(Bassani-Sternberg and Gfeller, 2016a)	MEL	1 patient	6	1.43	<0.01 ^B^	1	NetMHCpan 2.8
(Goh et al., 2016)	MCC	49 patients	4	0.09	N/A	1	NetMHC 3.4
(Gros et al., 2016)	MEL	3 patients	6	0.03	0.55 ^T^	2	IEDB
(Hugo et al., 2016)	MEL	38 patients	12	0.06	N/A	3	NetMHCpan 2.8, NetMHCIIpan 3.0
(Kalaora et al., 2016)	MEL	1 patient	6	5.30	<0.01 ^B^	1	NetMHCpan 2.8
(Karasaki et al., 2016)	NSCLC	15 patients	6	0.62	N/A	1	NetMHCpan 2.8
(Löffler et al., 2016)	CHOL	1 patient	6	3.68	0 ^B^	2	NetMHC 3.4, NetMHCpan 2.8, SYFPEITHI
(Strønen et al., 2016)	MEL	3 patients	1	0.05	0.19 ^T^	4	NetChop, NetMHC 3.2, NetMHCpan 2.0
(Anagnostou et al., 2017)	NSCLC	10 patients	6	0.76	<0.01 ^T^	4	SYFPEITHI, NetMHCpan, NetCTLpan
(Chang et al., 2017)	PED	540 patients	6	0.42	N/A	2	NetMHCcons 1.1
(Karasaki et al., 2017)	NSCLC	4 patients	6	0.20	N/A	2	NetMHCpan 2.8
(Kato et al., 2017)	BRCA	5 patients	6	0.47	N/A	2	NetMHC 3.4, NetMHCpan 2.8
(Miller et al., 2017)	MM	664 patients	6	0.16	N/A	3	NetMHC 4.0
(Ott et al., 2017)	MEL	6 patients	6	0.01	0.60 ^T^	3	NetMHCpan 2.4
(Sahin et al., 2017)	MEL	13 patients	10	0.02	0.60 ^T^	2	IEDB 2.5 (MHC class I & II)
(Zhang et al., 2017)	BRCA	3 patients	6	0.01	0.16 ^T^	3	NetMHC 3.2
(Kalaora et al., 2018)	MEL	15 patients/cell lines	6	9.57	0.15 ^T^	2	NetMHCpan 3.0
(Kinkead et al., 2018)	PAAD	1 murine cell line	2	0.27	0.16 ^T^	2	NetMHC 3.2/3.4, NetMHCpan 2.8
(Martin et al., 2018)	OV	1 patient	6	1.57	0,09 ^T^	2	NetMHCpan 2.4
(O’Donnell et al., 2018a)	OV	92 patients	6	0.02	N/A	2	NetMHCpan 2.8
(Sonntag et al., 2018)	PDAC	1 patient	10	2.00	0.75 ^T^	3	NetMHC, NetMHCIIpan 3.1, SYFPEITHI
(Thorsson et al., 2018)	various	8546 TCGA patients	6	0.74	N/A	2	NetMHCpan 3.0, pVAC-Seq 4.0.8
(Vrecko et al., 2018)	HCC	1 patient	3	0.05	0.15 ^T^	2	SYFPEITHI, IEDB (MHC class II)
(Wu et al., 2018)	various	7748 TCGA samples	100	1.18	N/A	1	NetMHCpan 4.0
(Bulik-Sullivan et al., 2019)	NSCLC	7 patients	6	0.10	0.08 ^T^	>4	EDGE
(Hilf et al., 2019)	GBM	10 patients	1	0.03	0.85 ^T^	3	IEDB 2.5
(Keskin et al., 2019)	GBM	8 patients	6	0.20	0.07 ^T^	3	NetMHCpan 2.4
(Koster and Plasterk, 2019)	various	10186 TCGA patients	1	0.02	N/A	2	NetMHC 4.0
(Liu et al., 2019)	OV	20 patients	12	0.15	0.24 ^T^	3	NetMHCpan 3.0, NetMHCIIpan 3.1
(Löffler et al., 2019)	HCC	16 patients	6	1.79	0 ^B^	2	NetMHC 4.0, NetMHCpan 3.0, SYFPEITHI
(Rosenthal et al., 2019)	NSCLC	164 samples/64 patients	6	0.86	N/A	2	NetMHC 4.0, NetMHCpan 2.8
(Schischlik et al., 2019)	PNMN	113 patients	6	2.53	0.66 ^B^	2	NetMHCpan

N/S means not specified. Cancer type abbreviations: adenocarcinoma (AC), breast cancer (BRCA), cholangiocarcinoma (CHOL), colorectal cancer (CRC), glioblastoma (GBM), gastrointestinal cancer (GIC), hepatocellular carcinoma (HCC), merkel cell carcinoma (MCC), melanoma (MEL), multiple myeloma (MM), non-small cell lung cancer (NSCLC), ovarian cancer (OV), pancreatic ductal adenocarcinoma (PDAC), pediatric cancers (PED), Ph-negative myeloproliferative neoplasms (PNMN), prostate adenocarcinoma (PRAD), sarcoma (SARC) and uterine corpus endometrial cancer (UCEC). ^T^ indicates experimentally confirmed T cell responses (e.g., IFNγ ELISPOT), ^B^ indicates experimentally confirmed major histocompatibility complex (MHC) binding (e.g., mass spectrometric [MS] of eluted peptides), and N/A indicates that no experimental validation was done. Features are mutated peptide binding prediction, wild-type peptide binding prediction, gene expression, sequence-based features like sequence similarity scores, and immunogenicity predictions. If available, version information of algorithms is included.

Not surprisingly, most publications investigated cancer types known for high mutation loads, such as non-small cell lung carcinoma and melanoma, but glioblastoma and chronic lymphocytic leukemia were also shown to harbor neoantigens identified by neoepitope prediction (Rajasagi et al., 2014; Hilf et al., 2019; Keskin et al., 2019). Regarding mutation types, the focus clearly lies on single nucleotide variants (SNVs) considering their abundance in tumors above all other types of mutation, their comparatively easy detection by mutation calling software and easier computational generation of mutated and wild-type peptide sequences (Bailey et al., 2018; Ellrott et al., 2018). However, larger indels, frameshifts, and other more complex mutation types can be the source of more neoepitopes that are also less similar to self and thus highly interesting immunotherapeutic targets. More recent studies from Kahles et al., Koster et al., and Schischlik et al. investigated these types of mutation, benefitting from improvements on sequencing and mutation calling techniques (Kahles et al., 2018; Koster and Plasterk, 2019; Schischlik et al., 2019). Nevertheless, identification of cancer-specific mutation remains a critical step in every neoepitope identification pipeline and the number of mutations obtained varies greatly dependent on the software and thresholds employed (Tran et al., 2015; Karasaki et al., 2017).

The focus of most publications lies on MHC class I presented neoepitopes that can be detected by CD8^+^ T cells. MHC class I prediction algorithms are more commonly used but there is clear evidence that MHC class II mediated CD4^+^ T cell responses play a major role in neoantigen immune responses and thus should also be considered for neoepitope detection. (Linnemann et al., 2014; Kreiter et al., 2015; Tran et al., 2015; Hugo et al., 2016; Ott et al., 2017; Reuben et al., 2017; Sahin et al., 2017; Sonntag et al., 2018; Vrecko et al., 2018).

All studies, except Koster et al., who investigated 10-mers only, looked at peptides with a length of 8–10 or 8–11 amino acids or just at 9-mers alone, which are the majority of peptides presented by MHC class I (Trolle et al., 2016). Most studies also relied on matching HLA types for the samples used, often determined by one of the following HLA typing algorithms: ATHLATES, HLAminer, OptiType, PHLAT, POLYSOLVER, and seq2HLA (Boegel et al., 2012; Warren et al., 2012; Liu et al., 2013; Szolek et al., 2014; Shukla et al., 2015; Bai et al., 2018). In contrast, Wu et al. made predictions based on the 100 most frequent HLA alleles in their dataset and Wood et al. based on the general 145 most frequent alleles (Wood et al., 2018; Wu et al., 2018). Whether or not such approaches yield substantial information gain is a debatable issue since most immunogenic mutations are highly individual and restricted by a patient’s individual HLA type (Marty et al., 2017; McGranahan et al., 2017; Rosenthal et al., 2019). HLA-A*02:01 has been extensively studied since it is the most common allele in Caucasian populations and therefore was exclusively used by Segal et al. for their analysis (Segal et al., 2008). Since predictions for A*02:01 still belong to the best performing group and can be more easily validated compared to other alleles due to established *in vitro* protocols and reagents, Carreno et al., Spranger et al., Strønen et al., van Gool et al., and Hilf et al. also only used A*02:01 for their predictions and the studies that carried out experimental validation accomplished high confirmation of predicted neoepitopes (Carreno et al., 2015; van Gool et al., 2015; Spranger et al., 2016; Strønen et al., 2016; Hilf et al., 2019). Similarly, Koster et al. only used A*02:01 for an unfiltered TCGA dataset although they did not perform experimental validation. Similar to Wood et al., they did not use HLA typing information for TCGA samples, which has been generated but can only be obtained by applying for access to restricted data (Shukla et al., 2015; Charoentong et al., 2017; Marty et al., 2017).

**Figure 3:**
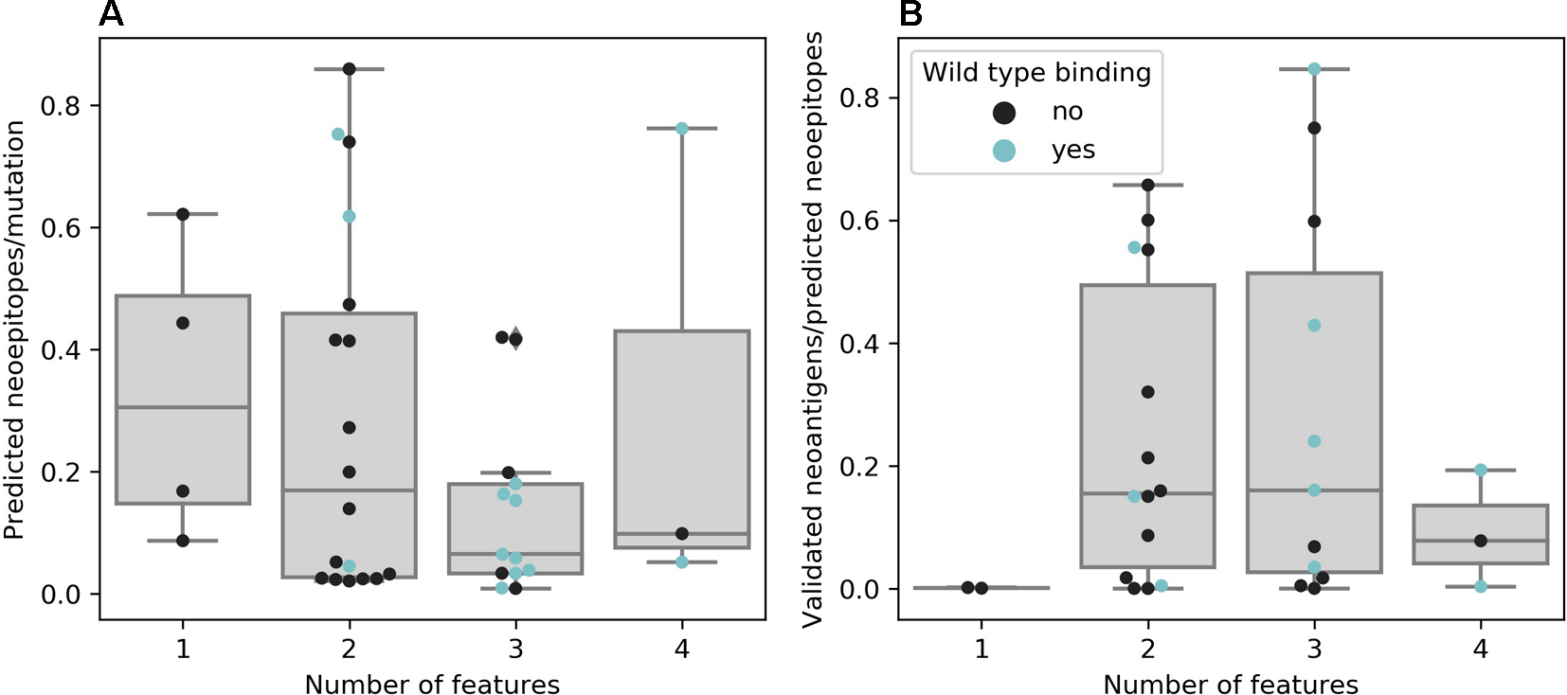
**(A)** Neoepitopes per mutation grouped by the number of features used for neoepitope selection. Data based on publications that offered comparable data, e.g., not obviously counting a neoepitope predicted to be presented by multiple major histocompatibility complexes (MHCs) multiple times (n = 38). **(B)** Ratio of confirmed to predicted neoepitopes grouped by the number of features used for neoepitope selection. Data based on publications that experimentally validated all predicted neoepitopes (n = 30)

For most studies, algorithms from the NetMHC family were chosen as they are widely known and represent the state-of-the-art prediction methods for binding of a peptide to a given MHC molecule. Van Allen et al. showed that out of 17 validated neoantigens, 14 passed the 500 nM standard threshold, indicating high sensitivity (van Buuren et al., 2014). However, only a handful of the predicted binders will also be recognized by T cells, which requires additional filtering or prediction improvement (Anonymous, 2017). Indeed, using more filtering criteria leads to fewer predicted neoepitopes per mutation, as seen in **Figure 3A**, although the false negative rate remains unknown. Only a few publications rely on predicting the binding affinity of mutated peptides alone and most use at least one additional threshold criterion, of which gene expression as a premise for antigen recognition is the most common. As RNA-Seq data was not available for Anagnostou et al., Le et al. and Reuben et al., they used TCGA expression data as a proxy to further filter the mutations to test for immunogenicity. Binding of the wild-type peptide was also considered by some studies, but not always used for filtering. Duan et al. proposed a “differential agretopicity index” (DAI), which is the difference between the predicted mutated and wild-type binding affinity, to use as a filtering criterion for neoepitope prediction. Although it yielded promising results based on their mouse data, it seemed less reliable in further investigations by Bjerregaard et al. and Koşaloğlu-Yalçın et al. using human data (Duan et al., 2014; Bjerregaard et al., 2017b; Koşaloğlu-Yalçın et al., 2018). In another study by Ghorani et al., DAI was more predictive for immune infiltration in melanoma and lung cancer compared to neoantigen or mutation load, suggesting that while some neoepitope responses might be enhanced by a reduced cross-reactivity potential, there are also many validated neoantigens whose wild-type counterparts are predicted to bind comparably strong (Ghorani et al., 2018; Koşaloğlu-Yalçın et al., 2018).

There is evidence that taking more than one feature into account promises greater success for experimentally validating predicted neoepitopes (see **Figure 3B**). However, the results of experimental validation are dependent on the sensitivity of the technique used and the reactivity of neoantigen-specific TILs can additionally be hampered by other factors, such as tumor immune suppression or T cell exhaustion (Anonymous, 2017; Bulik-Sullivan et al., 2019).

Some studies chose a quantitative approach, mostly linking neoepitope load and survival (Brown et al., 2014; Rizvi et al., 2015; Miller et al., 2017; Ghorani et al., 2018). It has to be mentioned that neoepitope load and mutational burden are usually highly correlated (Pearson r = 0.89 based on 38 publications with less than 1 neoepitope per mutation from **Table 1**) and although it can be assumed that an increased survival is linked to the immunogenicity of mutations, quantifying predicted neoepitopes does not necessarily transport more information than mutation burden alone (Nathanson et al., 2017). There are, however, also studies that correlated survival with neoepitopes but not mutational burden or found contradictory results depending on patient cohorts (Snyder et al., 2014; Ghorani et al., 2018).

Among well-described approaches for neoepitope identification based on affinity binding prediction algorithms, there are also pipelines available that automate all analytic steps and rank potential neoepitopes based on peptide affinity prediction and other features (see **Table 2**). They differ greatly as to their properties and outputs, thus offering choices depending on research questions and dataset sizes. Their availability demonstrates how important neoepitope prediction has become as an application for binding affinity prediction algorithms.

**Table 2:** Neoepitope prediction pipelines based on mutation data input. Additional features are cancer driver status of the mutated gene used by MuPeXI; differential agretopicity index (DAI), sequence-based immunogenicity score, and more used by Neopepsee; DAI, cleavage, and stability prediction used by pVACtools.

	MuPeXI	CloudNeo	Neopepsee	pVACTools
**Algorithms**	NetMHCpan	NetMHCpan	NetCTLpan, IEDB Bayes classifier	8 MHC class I predictors 4 MHC class II predictors
**Input**	VCF gene expression TSV	VCF BAM	VCF RNA-Seq FASTQ	VCF BAM (RNA and DNA)
**HLA typing**	user input	integrated	user input or integrated	user input or integrated
**Mutation types**	SNVs indels frameshifts	SNVs	SNVs	SNVs indels fusions (additional input)
**Wild type peptide**	yes	yes	yes	yes
**Gene expression**	yes (optional)	no	yes	yes
**Additional features**	yes	no	yes	yes
**Availability**	local, webserver	cloud	local	local
**Reference**	(Bjerregaard et al., 2017a)	(Bais et al., 2017)	(Kim et al., 2018)	(Hundal et al., 2019)

Since a variety of different neoepitope identification approaches exist and it is not clear which features are predictive for immunogenicity, Koşaloğlu-Yalçın et al. and Kim et al. integrated and compared features additional to the standard MHC binding affinity by either comparing areas under the curve of receiver operating characteristics or evaluating feature importance derived from trained classifiers (Kim et al., 2018; Koşaloğlu-Yalçın et al., 2018). Both studies found that binding affinity prediction performs best or is the most informative feature. This is not surprising for viral epitopes constituting a major part of data on which most prediction algorithms are trained nor for neoantigens from literature mainly selected by predicted binding affinity, which introduces a bias toward this feature. It still remains unclear how many potential neoantigens are not detected because their binding affinity is predicted to lie beyond thresholds. An approach avoiding this bias has been proposed by Bulik-Sullivan et al. (Bulik-Sullivan et al., 2019). Like the most recent generation of neural network binding prediction algorithms, they developed a deep learning neural network trained on MS data, but apart from improved peptide sequence modeling, they also included features unrelated to the pMHC interaction, namely, quantified gene expression, flanking sequence, and protein family. Although their model is currently limited to HLA alleles of the training data, the approach demonstrated an increased performance of neoepitope discovery over peptide binding prediction and can also be expanded to MHC class II presented antigens.

### Cross-Reactivity Assessment

A major challenge for immunotherapies introducing TCRs into patient recipient T cells is the choice of safe target antigens. If an engineered TCR-T cell cross-reacts with self-antigens in healthy tissue, the side-effects can be devastating. Possible TCR toxicity scenarios can be generally divided into on-target and off-target toxicities. On-target toxicities include all aspects of a specific target antigen or epitope expression that lead to an unintentional TCR-mediated destruction of healthy tissues. An example of on-target toxicity is melanocyte destruction, hearing loss, and retina infiltration mediated by MART1-targeting TCR-T cells relating to the same epitope in all cases (Johnson et al., 2009).

Off-target toxicities, in contrast, can appear by unexpected recognition of alternative epitopes that contain amino acid exchanges (mismatches) compared to the known epitope sequence. In rare cases, these mismatched peptides are presented identically on corresponding MHC molecules and are recognized equally well by deployed TCRs.

Targeting epitope sequences of proteins originating from highly homologous family members can cause unforeseen tissue damage as exemplified by the study performed by Morgan et al. (Morgan et al., 2013). Using autologous anti-MAGEA3 TCR-T cells, adoptive transfer led to severe neurotoxicity in several patients. The MAGEA3-specific TCR used in this clinical trial also recognized a MAGEA12, which was retrospectively found to be expressed in the brain. In the Linette et al. study, clinicians adoptively transferred MAGEA3-TCR-modified lymphocytes that also recognized an alternative epitope derived from the protein titin, causing fatal heart failure in two patients (Linette et al., 2013). Each of these examples underline the importance and need of comprehensive preclinical target and TCR analysis to prevent potential adverse events at later stages of clinical development.

With Expitope, we presented the first web server for assessing epitope sharing when evaluating new potential target candidates (Haase et al., 2015). Based on predictions for proteasomal cleavage, TAP transport, and MHC class I binding affinity, Expitope lists peptides with a given number of mismatches including the original target peptide. For these peptides, which are linked to genes by transcripts, the expression values in various healthy tissues, representing all vital human organs, are extracted from RNA-Seq data. However, transcript abundance only indirectly indicates protein expression. Meanwhile, proteome-wide human protein abundance data has become available and now facilitates a more direct approach for the prediction of potential cross-reactivity. The development of a new version 2.0 of Expitope, which computes all possible, naturally occurring epitopes of a peptide sequence and the corresponding cross-reactivity indices using both protein and transcript abundance levels weighted by a proposed hierarchy of importance of various human tissues, should help addressing this issue (Jaravine et al., 2017a). Cross-reactivity potential can also be assessed by calculating structural similarities between pMHC complexes obtained by molecular docking (Antunes et al., 2010) and by clustering pMHC complexes based on their electrostatic properties and the accessible surface area (Mendes et al., 2015). A comprehensive review by Baker et al. (2012) is covering these aspects in great detail.

## TCR Binding Prediction

**Figure 4:**
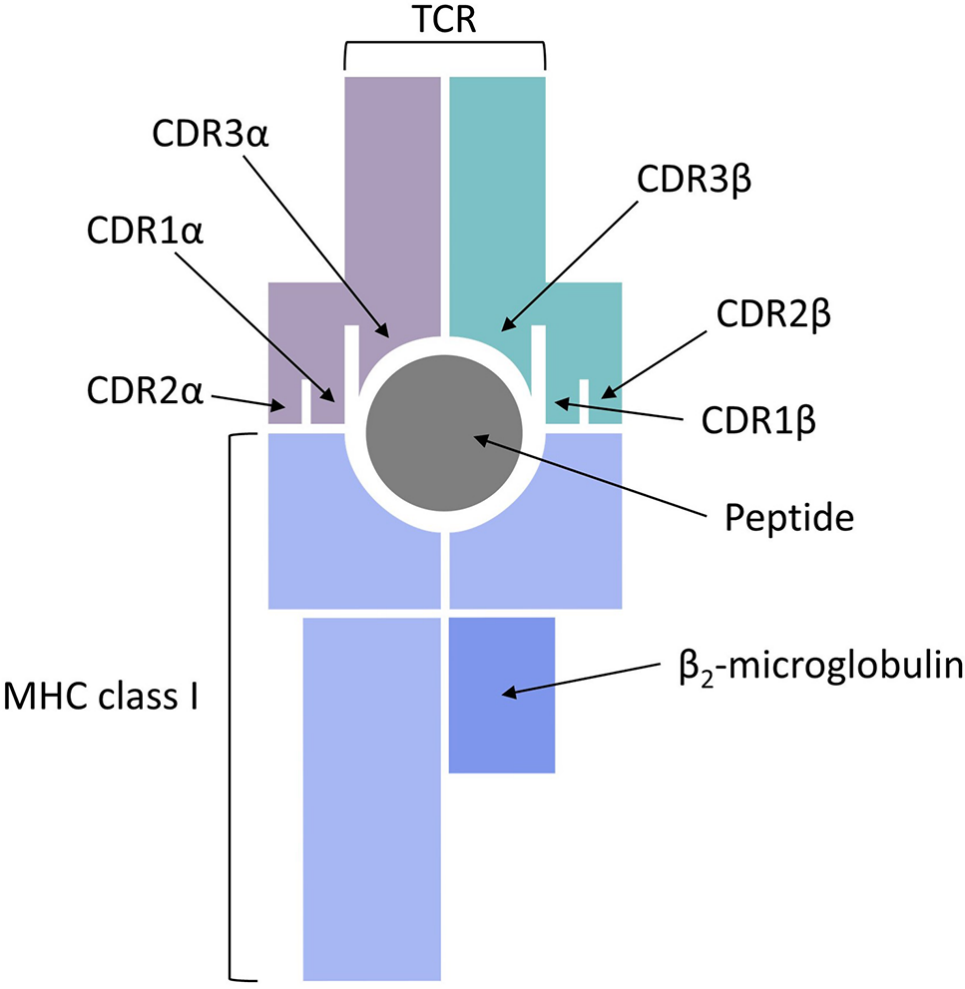
T cell receptor (TCR) binding to a peptide presented by major histocompatibility complex (MHC) class I.

The final piece of the epitope recognition puzzle is the interaction of the pMHC complex with the TCR, which represents a very difficult problem for modeling studies and sequence-based predictions. One reason for that is the complex and noncontiguous nature of the interaction interface, with the CDR1 and CDR2 regions of the TCR α and β chains making contacts with the MHC class I molecule and the CDR3 regions directly interacting with the bound peptide (see **Figure 4**). Another major hurdle in predicting TCR recognition is the scarcity of experimentally confirmed TCR complementarity determining regions and the sequences of their respective binding partners on the pMHC complex. For example, one of the first feasibility studies of CDR3 sequence patterns was only based on two immunogenic HIV peptides (De Neuter et al., 2018). An additional complication is posed by the fact that repertoire sequencing combined with immune assays determines antigen-specific clonotypes, but does not yield negative controls, i.e., validated pairs of CDRs and pMHC complexes that do not bind each other.

CDR3β chains appear to always be in contact with the antigen bound to the MHC class I molecule, whereas the direct contact of CDR3α chains to the peptide is not always required (Glanville et al., 2017). The involvement of short linear stretches of CDR3β sequence in peptide-TCR interactions creates the opportunity to cluster TCRs in groups of common specificity (Dash et al., 2017; Glanville et al., 2017) and also serves as the basis for developing specialized algorithms for sequence-based prediction of pMHC/TCR binding. Two recent publications addressed this problem from two completely different perspectives. Jurtz et al. presented a proof of concept study, in which they predicted TCR interactions with their cognate HLA-A*02:01-presented peptide targets (Jurtz et al., 2018). A machine learning approach, called NetTCR, was trained on 8,920 TCRβ CDR3 sequences and 91 cognate peptide targets obtained from IEDB and from the immune assay data published by Klinger et al. (2015). A dataset of negative interactions was assembled by randomly matching TCR and peptide pairs. The NetTCR project in its current form is limited to a small number of peptides and it does not consider CDR1/CDR2 interactions with the MHC molecules or CDR3α sequences, but it is an important step forward because it demonstrates that TCR recognition of pMHCs is specific enough to be captured by sequence-level prediction tools.

Ogishi and Yotsuyanagi exploited the existence of immunodominant epitopes, which are targeted by the adaptive immune system in different individuals and would therefore be expected to exhibit some prominent features that make them especially prone to be recognized by T cells (Ogishi and Yotsuyanagi, 2019). The idea behind their repertoire-wide TCR-epitope contact potential profiling is that intermolecular contacts between relevant portions of the epitope and the TCR CDR3β region that closely resemble the contact structure of the interactions involving immunodominant peptides would be more likely to be immunogenic. To quantitatively assess the interaction affinity, they used physicochemical properties of amino acids and an energetic potential, calculated as the sum of all pairwise contact potentials for individual amino acids. The latter were obtained from several previously published amino acid contact potential scales, available from the AAINDEX database (Kawashima et al., 2007). These features were converted to immunogenicity scores using machine learning. It should be noted that the knowledge-based potentials, derived from crystal structures of proteins and protein complexes, reflect either intramolecular interactions driving protein folding and stability or contacts at protein interfaces and may only be a coarse approximation of peptide-TCR interactions. Yet, Ogishi and Yotsuyanagi demonstrated that the most informative contact-based and property-based features strongly correlate with experimentally measured TCR-peptide affinities.

**Figure 5:**
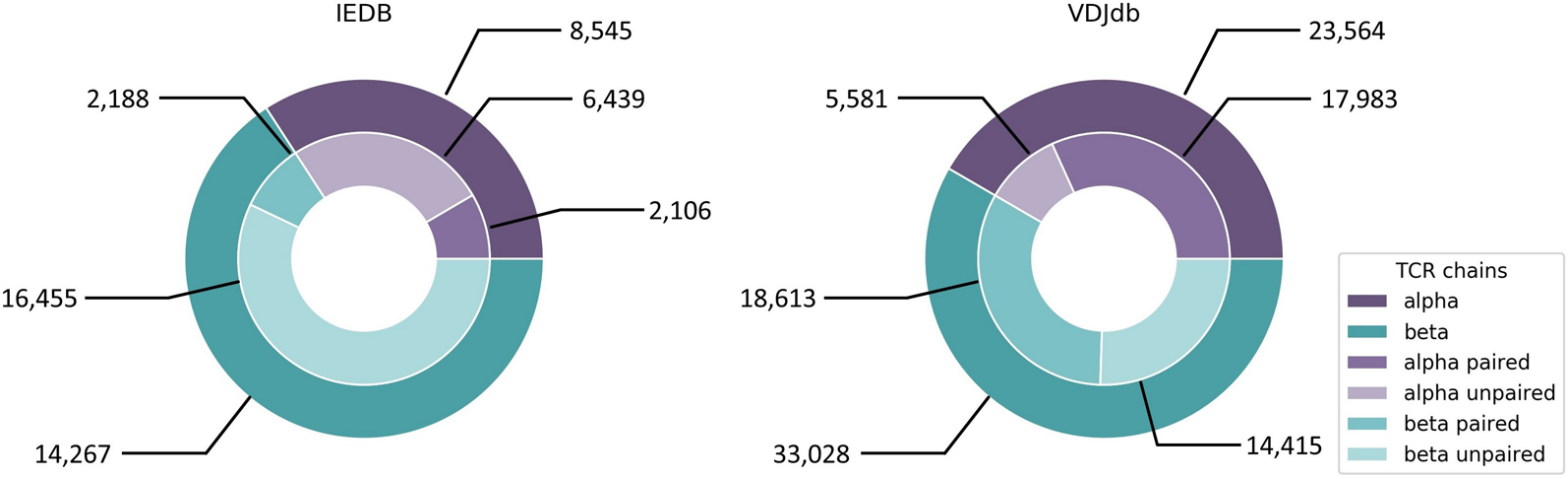
IEDB and VDJdb contents of CDR3α and CDR3β sequences of human origin. IEDB contains 386 unique epitopes linked to CDR3α sequences and 426 unique epitopes linked to CDR3β sequences. For VDJdb there are 93 and 177 unique epitopes, respectively. IEDB data was downloaded from https://www.iedb.org on September 30th, 2019 with the following query parameters: Current Filters: No B cell assays, No major histocompatibility complex (MHC) ligand assays, Restriction Type: Class I, Host: Homo sapiens (human). VDJdb data was taken from https://vdjdb.cdr3.net/overview (last updated on August 7th, 2019).

Both approaches by Jurtz et al. and Ogishi and Yotsuyanagi are solely based on CDR3β chains and do not incorporate CDR3α sequence information. This is due to the fact that most datasets and databases such as IEDB and VDJdb did, until recently, consist mainly of CDR3β sequences (**Figure 5**) derived from bulk sequencing (Shugay et al., 2018; Vita et al., 2018), since identifying functional TCR pairing in repertoire data is technically challenging (Holec et al., 2018). Single cell sequencing eliminates this problem and a large dataset has just been added to VDJdb, which is, however, dominated by only few epitopes and HLA alleles. Another problem regarding TCR-epitope data is the lack of true negative datasets and the inclusion of cross-reactivity information, since many TCRs are able to recognize more than one epitope, which has been elaborated in section “Cross-reactivity assessment.” For this reason, pMHC/TCR binding prediction would also add valuable information to the detection of potential cross-reactivity for clinical candidate TCRs.

Further light on the details of pMHC/TCR interactions can be shed by molecular dynamics simulations. This entails understanding the role of hydrogen bonds, hydrophobic contacts, and interactions with the solvent in determining the specificity and cross-reactivity of each individual complex and proposing specific models of TCR engagement with the CDR1, CDR2, and CDR3 regions (Cuendet et al., 2011). Moreover, molecular modeling can help to compare the surface morphology between the complexed wild-type and mutated peptides and their relationship with immunogenicity (Park et al., 2013) and can also help to predict affinity-enhancing TCR mutations (Malecek et al., 2014). In cases where three-dimensional structures are not yet available, accurate models of pMHC/TCR complexes can be obtained by homology modeling (Zoete et al., 2013; Lanzarotti et al., 2019). Finally, a number of both rigid and flexible pMHC/TCR docking protocols have been proposed, which, in many cases, are able to produce accurate complex models starting from unbound structures (Pierce and Weng, 2013).

## Conclusion and Outlook

Machine learning has become an indispensable tool for immunotherapeutic applications over the last decades. The established core method is peptide binding affinity prediction and thus target identification for TCR-T therapy or personalized neoantigen vaccination. The constant evolution of available training data as well as machine learning techniques, building on growing computational power, has improved the quality of binding affinity predictions. Focus has been on CD8^+^ cytotoxic T cells, but the substantial role of CD4^+^ T cells is increasingly gaining attention and efforts are made to also improve predictions for MHC class II presented epitopes, which poses a more challenging task compared to MHC class I binding due to the larger variety in peptide length and the open binding groove (Brown et al., 1993).

Additional challenges which can be tackled by machine learning remain. Immunogenicity is still an elusive aim for prediction tools, especially when it comes to personalized therapies relying on neoepitope identification. This is owed to the fact that patient immune systems and tumors undergo a process of mutual influence and therefore are highly individual and heterogeneous. The identification of features derived from the immune system that affect T cell recognition of individual epitopes within a tumor could be the key toward more reliable personalized immunotherapy predictions, thereby opening the process to a broader number of patients. Although neoantigens are currently in the focus of cancer immunotherapy, the detection of shared tumor antigens beyond coding DNA regions remains necessary since not all tumors harbor enough immunogenic mutations and the creation of potent TCRs for individual patients is currently impossible. Another challenge, which can be tackled with the help of ongoing data acquisition, is TCR binding prediction. Being able to reliably predict which TCR will recognize which epitope is extremely valuable not only for target epitope identification for TCR-T therapies, but also especially for TCR safety assessment, since it can speed up the process of selecting TCRs for the clinic by reducing *in vitro* screening of TCR candidates.

As the TCR-T adoptive immunotherapy community grows and data on the impact of sequence variations in both TCR alpha and beta chains on peptide fine specificity, sensitivity of peptide-MHC recognition and TCR cross-reactivity for partially mismatched epitopes emerge, artificial intelligence in the form of machine learning will be critical to advance understanding of pMHC/TCR interactions for many types of antigen and many different HLA allotypes. In particular, these issues will become additionally relevant as this form of immunotherapy is developed for patient populations worldwide. High-throughput TCR discovery platforms, yielding TCR sequence information from natural repertoires of T cells or through TCR mutational analyses, coupled with functional assessment of peptide variants as a means to assess cross-reactivity, offer many opportunities to continually improve understanding of pMHC/TCR interactions that will not only advance the cause of basic science but also help to meet medical needs for patients with cancer, infectious diseases or autoimmunity, where it is envisioned that TCR-Ts have the potential to provide improved therapies worldwide.

In particular, the push to couple TCR sequence data with neoantigen recognition for single patients through analysis of individual tumor samples in order to develop more potent cancer vaccines or TCR-T immunotherapies has already fostered strong collaborations and commercial endeavors to advance the interplay of machine learning and TCR recognition. While it currently seems daunting to imagine how the enormous and fast flow of information now emerging from many sources can be accessed and assembled to rapidly support the broader needs for personalized patient-individualized TCR-based immunotherapies, this review summarizes the challenges as well as the substantial progress that has already been achieved in defining some of the most relevant parameters in the complex cell biology of antigen processing and presentation and pMHC interactions with TCRs that lead to successful immune recognition. Important gaps have also been defined, alerting the community to the types of control data that may already exist in many laboratories, or could be collected, that would help in the refinement of prediction tools to achieve better results in the future. Increased interest and collaborative efforts of machine learning and HLA and TCR specialists will certainly foster further developments to support the rapidly expanding field of T cell-based immunotherapy of high medical relevance.

With the support of bioinformatic tools and improved prediction algorithms, immunotherapy holds the potential to become more precise, more personalized, and more effective than current cancer treatments—and potentially with fewer side effects.

## Author Contributions

AM, SR, MW, DS, and DF all contributed to the writing and all approved the content of this review article.

## Conflict of Interest

AM, SR, and MW are employees and DS is a Managing Director of Medigene Immunotherapies GmbH, a subsidiary of Medigene AG, Planegg, Germany.

The remaining author declares that the research was conducted in the absence of any commercial or financial relationships that could be construed as a potential conflict of interest.
